# Predictive Performance of Machine Learning–Based Models for Poststroke Clinical Outcomes in Comparison With Conventional Prognostic Scores: Multicenter, Hospital-Based Observational Study

**DOI:** 10.2196/46840

**Published:** 2024-01-11

**Authors:** Fumi Irie, Koutarou Matsumoto, Ryu Matsuo, Yasunobu Nohara, Yoshinobu Wakisaka, Tetsuro Ago, Naoki Nakashima, Takanari Kitazono, Masahiro Kamouchi

**Affiliations:** 1 Department of Health Care Administration and Management Graduate School of Medical Sciences Kyushu University Fukuoka Japan; 2 Department of Medicine and Clinical Science Graduate School of Medical Sciences Kyushu University Fukuoka Japan; 3 Biostatistics Center Graduate School of Medicine Kurume University Kurume Japan; 4 Big Data Science and Technology Faculty of Advanced Science and Technology Kumamoto University Kumamoto Japan; 5 Center for Cohort Studies Graduate School of Medical Sciences Kyushu University Fukuoka Japan; 6 Medical Information Center Kyushu University Hospital Fukuoka Japan

**Keywords:** brain infarction, outcome, prediction, machine learning, prognostic score

## Abstract

**Background:**

Although machine learning is a promising tool for making prognoses, the performance of machine learning in predicting outcomes after stroke remains to be examined.

**Objective:**

This study aims to examine how much data-driven models with machine learning improve predictive performance for poststroke outcomes compared with conventional stroke prognostic scores and to elucidate how explanatory variables in machine learning–based models differ from the items of the stroke prognostic scores.

**Methods:**

We used data from 10,513 patients who were registered in a multicenter prospective stroke registry in Japan between 2007 and 2017. The outcomes were poor functional outcome (modified Rankin Scale score >2) and death at 3 months after stroke. Machine learning–based models were developed using all variables with regularization methods, random forests, or boosted trees. We selected 3 stroke prognostic scores, namely, ASTRAL (Acute Stroke Registry and Analysis of Lausanne), PLAN (preadmission comorbidities, level of consciousness, age, neurologic deficit), and iScore (Ischemic Stroke Predictive Risk Score) for comparison. Item-based regression models were developed using the items of these 3 scores. The model performance was assessed in terms of discrimination and calibration. To compare the predictive performance of the data-driven model with that of the item-based model, we performed internal validation after random splits of identical populations into 80% of patients as a training set and 20% of patients as a test set; the models were developed in the training set and were validated in the test set. We evaluated the contribution of each variable to the models and compared the predictors used in the machine learning–based models with the items of the stroke prognostic scores.

**Results:**

The mean age of the study patients was 73.0 (SD 12.5) years, and 59.1% (6209/10,513) of them were men. The area under the receiver operating characteristic curves and the area under the precision-recall curves for predicting poststroke outcomes were higher for machine learning–based models than for item-based models in identical populations after random splits. Machine learning–based models also performed better than item-based models in terms of the Brier score. Machine learning–based models used different explanatory variables, such as laboratory data, from the items of the conventional stroke prognostic scores. Including these data in the machine learning–based models as explanatory variables improved performance in predicting outcomes after stroke, especially poststroke death.

**Conclusions:**

Machine learning–based models performed better in predicting poststroke outcomes than regression models using the items of conventional stroke prognostic scores, although they required additional variables, such as laboratory data, to attain improved performance. Further studies are warranted to validate the usefulness of machine learning in clinical settings.

## Introduction

### Background

Despite receiving the best available treatment, patients who have had a stroke may still experience disability or, in some cases, even face the risk of death [1,2]. Stroke clinicians try to predict patients’ outcomes as accurately as possible because accurate prognoses are a prerequisite for therapeutic decisions. Various stroke prognostic scores have been developed to support clinicians in predicting poststroke outcomes [3-8]. Nevertheless, prognostic scores have some disadvantages: generally, they limit the number of variables for ease of use at the bedside, and their validity needs to be reappraised over time, as the scoring criteria may become outdated with rapid progress in stroke care [9].

Meanwhile, recent advances in information technology have enabled the collection of a large amount of health information on individual patients [10,11]. Machine learning is considered a promising tool for improving the prediction accuracy of clinical outcomes for individual patients with stroke because of the ability of machine learning to deal with large and complex data [12-24].

However, several papers questioning the incremental value of machine learning have recently been published [25-27]. One study reported that machine learning algorithms did not perform better than traditional regression models for making prognoses in traumatic brain injury and recommended replicating studies in fields other than traumatic brain injury to ensure the generalizability of the findings [26]. Hitherto, few studies have directly compared the performance of data-driven models developed using machine learning methods and regression models based on conventional stroke prognostic scores in the field of outcome prediction after ischemic stroke [19,20,23]. In addition, calibration has not been adequately addressed in previous studies, and model performance has primarily been evaluated based on its discriminative ability [18-20].

### Objectives

In this study, we aimed to examine whether machine learning can improve the predictive performance for poststroke outcomes beyond preexisting stroke prognostic scores. We also sought to elucidate the pattern of variables selected by machine learning algorithms to predict poststroke clinical outcomes. To this end, we analyzed the data of patients with acute ischemic stroke enrolled in a multicenter, hospital-based, prospective registry of stroke in Japan. We used 3 stroke prognostic scores, namely, Acute Stroke Registry and Analysis of Lausanne (ASTRAL) score [6], preadmission comorbidities, level of consciousness, age, and neurologic deficit (PLAN) score [7], and Ischemic Stroke Predictive Risk Score (iScore) [4,5], to create item-based regression models. We then compared the predictive performance of data-driven models developed using machine learning algorithms with that of item-based models in identical study populations. We also examined the explanatory variables used in data-driven models and compared them with the items of the conventional prognostic scores.

## Methods

### Ethical Considerations

The study protocol was approved by the institutional review boards of all hospitals (Kyushu University Institutional Review Board for Clinical Research: 22086-01; Kyushu Medical Center Institutional Review Board: R06-03; Clinical Research Review Board of Fukuokahigashi Medical Center: 29-C-38; Fukuoka Red Cross Hospital Institutional Review Board: 629; St Mary’s Hospital Research Ethics Review Committee: S13-0110; Steel Memorial Yawata Hospital Ethics Committee: 06-04-13; and Kyushu Rosai Hospital Institutional Review Board: 21-8). Written informed consent was obtained from all patients or their family members.

### Data Source

We used data from the Fukuoka Stroke Registry (FSR), a multicenter, hospital-based, prospective registry of patients with acute stroke. FSR enrolled patients with stroke hospitalized in 7 participating hospitals in Fukuoka, Japan, within 7 days of onset (University Hospital Medical Information Network Clinical Trial Registry: UMIN000000800). Details of the registry have been previously published [28,29]. In FSR, clinical data during routine stroke care in the hospitals were recorded along with baseline information on variables such as demographics, prior history, comorbidity, and functional level before stroke onset. The definitions of these variables have been previously described [28,29].

### Stroke Prognostic Scores

The conventional stroke prognostic scores were used for comparison against data-driven prediction models. In this study, we selected prognostic scores based on the following criteria: they are multiitem and point-based scores using demographic and clinical information, they were developed to predict short-term outcomes after ischemic stroke, and they were externally validated. Consequently, 3 stroke prognostic scores, the ASTRAL score [6], PLAN score [7], and iScore [4,5], were used for comparative analysis. Items of these preexisting stroke prognostic scores were used as explanatory variables in item-based models (Multimedia Appendix 1).

### Study Populations

FSR included 10,700 consecutive patients with acute ischemic stroke who were registered between June 2007 and May 2017. Ischemic stroke was diagnosed based on the sudden onset of a nonconvulsive and focal neurological deficit confirmed by brain imaging through computed tomography, magnetic resonance imaging, or both conducted upon admission. Of the 10,700 patients, 187 (1.7%) were lost to follow-up, and the remaining 10,513 (98.3%) were analyzed for 3 months post stroke.

**Figure 1:**
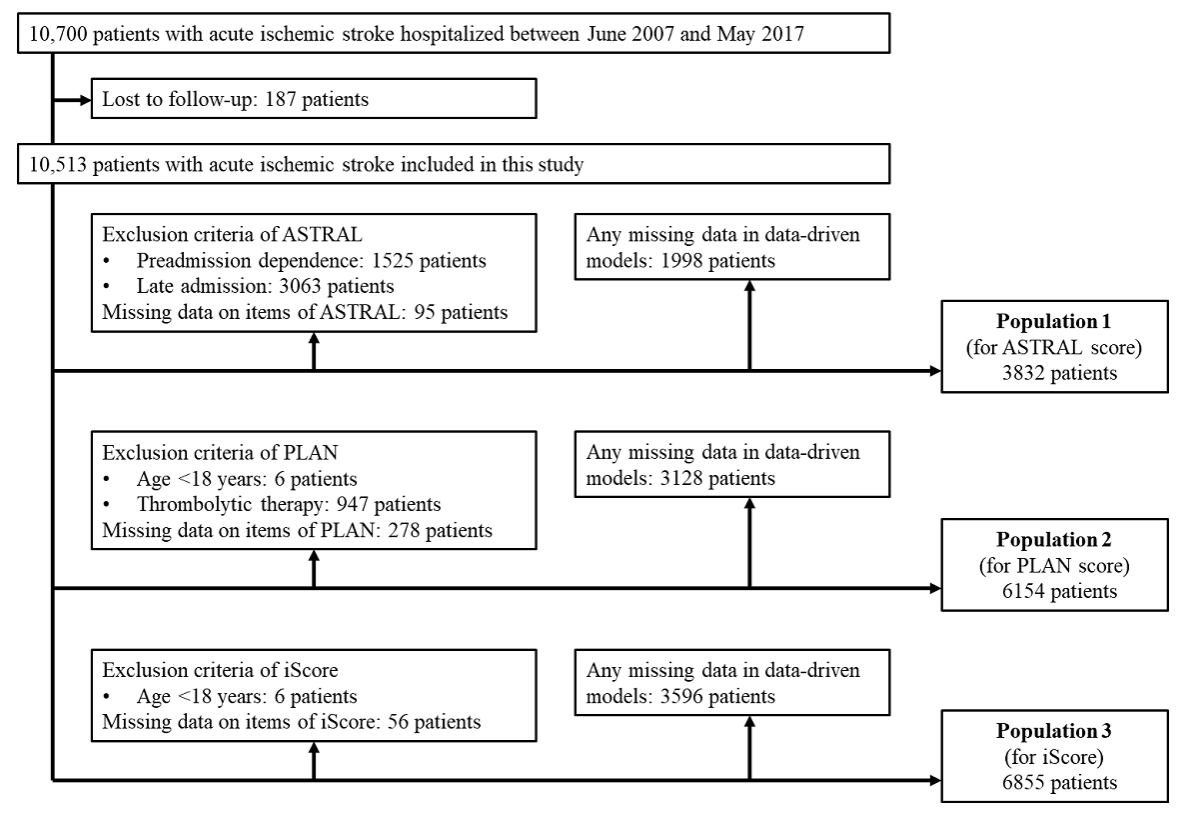
Flowchart for the selection of study patients. Study patients were selected according to the inclusion and exclusion criteria used in the original studies of 3 stroke prognostic scores: population 1 for the Acute Stroke Registry and Analysis of Lausanne (ASTRAL) score, population 2 for the preadmission comorbidities, level of consciousness, age, and neurologic deficit (PLAN) score, and population 3 for the Ischemic Stroke Predictive Risk Score (iScore). Patients with missing data on explanatory variables were excluded from the analyses of data-driven models to avoid the influence of list-wise deletion.

Study patients were selected according to the inclusion and exclusion criteria of preexisting stroke prognostic scores to make the study populations identical between the item-based and machine learning–based models (Multimedia Appendix 2). Furthermore, we limited the study to patients with complete data, ensuring there were no missing variables across all data points. This approach aimed to prevent further reduction in the number of analyzed patients owing to list-wise deletion in regression models. The frequency of missing data is shown in Multimedia Appendix 3. Consequently, population 1, population 2, and population 3 were included in the analysis for comparison with the ASTRAL score, PLAN score, and iScore, respectively. Figure 1 illustrates the patient selection in each population.

### Study Outcomes

The study outcomes were poor functional outcome and death at 3 months after stroke. Poor functional outcome was defined as a modified Rankin Scale score >2 at 3 months after stroke onset [30]. Death was defined as death from any cause within 3 months after stroke [30]. Interviewers on clinical outcomes were blinded to the patients’ backgrounds.

### Development of Predictive Models

We performed logistic regression analysis to develop item-based models using the predictors of the ASTRAL score, PLAN score, and iScore as explanatory variables (Multimedia Appendix 1). The predictors used in these models included age, time delay from onset to admission, stroke scale score, decreased level of consciousness, visual field defect, and abnormal glucose levels for the ASTRAL score; age, atrial fibrillation, congestive heart failure, cancer, preadmission dependence, decreased level of consciousness, leg weakness, arm weakness, and aphasia or neglect for the PLAN score; age, male sex, atrial fibrillation, congestive heart failure, renal dialysis, cancer, preadmission dependence, Canadian Neurological Scale score, stroke subtype, and abnormal glucose levels for the iScore. The categorization of predictors in the stroke prognostic scores was the same as that used in the original study for each score.

We used regularization methods (ridge regression [RR] and least absolute shrinkage and selection operator [LASSO] regression models) and ensemble decision tree models (random forest [RF] and Extreme Gradient Boosting [XGBoost]) for data-driven models based on machine learning algorithms [31-34]. All available variables were included in the development of data-driven models (Multimedia Appendix 3). The details of the model development are presented in Multimedia Appendix 4.

### Metrics of Model Performance

The discriminative ability of each model was evaluated using the area under the receiver operating characteristic curve (AUROC) and the area under the precision-recall curve (AUPRC). AUPRC was calculated because it is a useful performance metric for unbalanced data of infrequent outcome events, such as death [35].

The calibration of each model was assessed using a calibration plot. Calibration plots were obtained by plotting the predicted and observed probabilities of the clinical outcomes in the 10 risk groups estimated using each predictive model. The Brier score was also used to assess the overall performance. The Brier score is defined as 1/N ∑^N^_i=1_ (pi–ai)^2^, (0≤BS≤1), where pi is the predicted probability of the occurrence of an event ranging from 0 to 1, ai indicates the event with binary outcomes (1 for observed or 0 for not observed), and N is the number of samples.

### Validation and Comparison of Models

**Figure 2:**
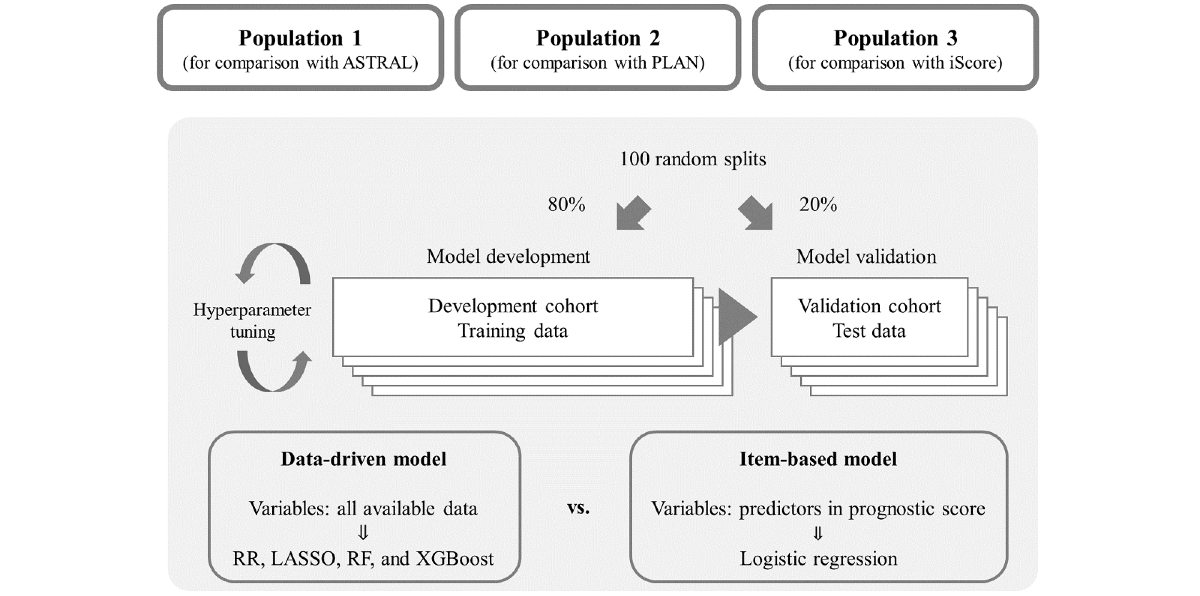
Schematic diagram of the development and validation of the predictive models. All patients were randomly split into 80% of the development cohort as training data and 20% of the validation cohort as test data, which was repeated 100 times. Among the data-driven models, predictive models were developed based on ridge regression (RR), least absolute shrinkage and selection operator regression (LASSO), random forest (RF), and Extreme Gradient Boosting (XGBoost) using all available data after hyperparameter tuning in the development cohort. Logistic regression was used with predictors of stroke prognostic scores in the item-based models. The predictive models were validated using the test data of the validation cohort. In each split, the training and test data were identical between the data-driven and item-based models. ASTRAL: Acute Stroke Registry and Analysis of Lausanne; PLAN: preadmission comorbidities, level of consciousness, age, and neurologic deficit.

We performed internal validation of item-based and data-driven models after 100 repeated random splits into 80% of the patients as a training set and 20% of patients as a test set (Figure 2). The parameters in the training set were optimally tuned via 10-fold cross-validation in the data-driven models. After 100 random splits, the predictive models were developed by logistic regression using the items of the stroke prognostic scores (item-based model) and by machine learning using all variables (data-driven model) in the training set. The developed item-based and data-driven models were validated in the test set. The data sets for both training and testing were identical for the item-based and data-driven models. The median and 95% CI of the performance metrics, that is, AUROC, AUPRC, and Brier score, were calculated for each model using the results of the 100 repeated random splits. To directly compare the performance of the item-based and data-driven models (RR, LASSO, RF, and XGBoost), we compared the AUROC, AUPRC, and Brier score of the data-driven models with those of the corresponding item-based model. We repeated the comparison 100 times and calculated the times that the AUROC, AUPRC, and Brier score of data-driven models were better than those of the corresponding item-based model among the 100 repetitions.

### Evaluation of the Contribution of Variables

We evaluated the importance of the variables used in the item-based and data-driven models. To assess the contribution of each predictor to the item-based regression model, we calculated the rate of times when the association between each variable and clinical outcomes was statistically significant (*P*<.05) after 100 random splits. In the machine learning models, the magnitude of variable importance was evaluated in identical populations after 100 random splits (Multimedia Appendix 4).

We calculated the AUROC of the XGBoost model using various types of variables to assess how the addition of explanatory variables improves the predictive performance of the data-driven model. First, we constructed a model with age, sex, National Institutes of Health Stroke Scale (NIHSS) score, and preadmission modified Rankin Scale score (model 1). Then, 5 models were developed by adding items relating to preadmission status to model 1 (model 2), items relating to clinical data on admission to model 2 (model 3), items relating to brain imaging data to model 3 (model 4), and items relating to laboratory data to model 4 (model 5).

### Statistical Analysis

We used the chi-square test, 2-tailed Student *t* test, or Mann-Whitney *U* test to compare the differences in baseline characteristics and clinical data, as appropriate [36]. Two-sided *P* values <.05 were considered statistically significant.

All statistical analyses were performed using the R statistical package (R Development Core Team). This study was conducted in accordance with the Transparent Reporting of a Multivariable Prediction Model for Individual Prognosis or Diagnosis (TRIPOD) initiative [37].

## Results

### Baseline Variables and Clinical Outcomes

The mean age of the 10,513 patients was 73.0 (SD 12.5) years, and 59.1% (6209/10,513) of the patients were men. At 3 months after stroke, a poor functional outcome was found in 1204 (31.4%) of 3832 patients in population 1, 2209 (35.9%) of 6154 patients in population 2, and 2540 (37.1%) of 6855 patients in population 3. Within 3 months after stroke onset, 3% (113/3832), 3.6% (219/6154), and 3.7% (255/6855) of the patients died in population 1, population 2, and population 3, respectively.

First, we investigated the differences in the predictors of preexisting point-based stroke prognostic scores among patients according to poststroke clinical outcomes. Consequently, almost all variables significantly (*P*<.05) differed depending on the 3-month functional outcome (Table 1) and 3-month survival status (Multimedia Appendix 5) in addition to the predictors used in preexisting prognostic scores.

**Table 1:** Baseline data according to functional outcome at 3 months.

				Overall (n=10,513)		mRS^a^ 0-2 (n=6405)		mRS 3-6 (n=4108)		*P* value	
**Demographics**											
	Age (y), mean (SD)		73.0 (12.5)		68.9 (12.0)		79.4 (10.4)		<.001		
	Men, n (%)		6209 (59.1)		4257 (66.5)		1952 (47.5)		<.001		
**Risk factors, n (%)**											
	Hypertension		8485 (80.7)		5138 (80.2)		3347 (81.5)		.11		
	Diabetes mellitus		3607 (34.3)		2236 (34.9)		1371 (33.4)		.11		
	Atrial fibrillation		2743 (26.1)		1173 (18.3)		1570 (38.3)		<.001		
	Smoking		2261 (23.1)		1717 (28.9)		544 (14.2)		<.001		
**Comorbid conditions, n (%)**											
	Congestive heart failure		919 (8.7)		423 (6.6)		496 (12.1)		<.001		
	Kidney disease on dialysis		332 (3.2)		171 (2.7)		161 (3.9)		<.001		
	Cancer		1552 (14.8)		774 (12.1)		778 (18.9)		<.001		
**Previous history, n (%)**											
	Previous myocardial infarction		505 (5.3)		242 (4.3)		263 (6.9)		<.001		
**Preadmission functional status**											
	Preadmission mRS, median (IQR)		0 (0-1)		0 (0-0)		1 (0-3)		<.001		
	Preadmission dependence (mRS score >1), n (%)		2366 (22.5)		364 (5.7)		2002 (48.7)		<.001		
**Onset-to-admission time, n (%)**											<.001
	≤1 h		943 (9)		490 (7.7)		453 (11)				
	≤3 h		1469 (14)		771 (12)		698 (17)				
	≤6 h		1141 (10.9)		644 (10.1)		497 (12.1)				
	≤24 h		3515 (33.4)		2090 (32.6)		1425 (34.7)				
	>24 h		3445 (32.8)		2410 (37.6)		1035 (25.2)				
**Stroke subtype, n (%)**											<.001
	Small vessel occlusion		2119 (20.2)		1724 (26.9)		395 (9.6)				
	Large artery atherosclerosis		1823 (17.3)		1006 (15.7)		817 (19.9)				
	Cardioembolism		2496 (23.7)		1054 (16.5)		1442 (35.1)				
	Other determined etiology		2146 (20.4)		1404 (21.9)		742 (18.1)				
	Undetermined		1929 (18.3)		1217 (19)		712 (17.3)				
**Neurological severity, median (IQR) or n (%)**											
	NIHSS^b^ score		3 (2-8)		2 (1-4)		8 (4-16)		<.001		
	Severe stroke (NIHSS score >10)		1938 (18.4)		291 (4.5)		1647 (40.1)		<.001		
**Neurological deficits, n (%)**											
	Decreased level of consciousness		3129 (30)		770 (12.1)		2359 (57.9)		<.001		
	Leg weakness		5394 (51.9)		2357 (37.2)		3037 (75)		<.001		
	Arm weakness		5634 (54.2)		2520 (39.7)		3114 (76.8)		<.001		
	Aphasia or neglect		2912 (27.9)		946 (14.9)		1966 (48.3)		<.001		
	Visual field defect		999 (9.6)		447 (7.0)		552 (13.6)		<.001		
**Physiological data, mean (SD)**											
	SBP^c^, mm Hg		86.6 (18.2)		87.9 (17.8)		84.6 (18.6)		<.001		
	DBP^d^, mm Hg		159.8 (29.3)		160.4 (28.6)		158.8 (30.3)		.01		
	BMI, kg/m^2^		22.8 (3.8)		23.5 (3.6)		21.7 (3.9)		<.001		
**Laboratory data, median (IQR)**											
	**Complete blood cell count**										
		WBC^e^ (10^3^/μL)	6.8 (5.6-8.4)		6.7 (5.5-8.2)		7.0 (5.7-8.9)		<.001		
		RBC^f^ (10^4^/μL)	436 (394-476)		449 (411-485)		416 (372-458)		<.001		
		Hematocrit (%)	40.1 (36.5-43.4)		41.1 (37.9-44.0)		38.2 (34.6-41.9)		<.001		
		Hemoglobin (g/dL)	13.5 (12.1-14.8)		14.0 (12.7-15.1)		12.8 (11.4-14.1)		<.001		
		Platelet (10^4^/μL)	20.2 (16.6-24.3)		20.6 (17.0-24.7)		19.5 (15.8-23.6)		<.001		
	**Liver function**										
		AST^g^ (U/L)	23 (19-29)		23 (19-29)		23 (19-30)		.001		
		ALT^h^ (U/L)	17 (12-24)		18 (13-25)		15 (11-22)		<.001		
		LDH^i^ (U/L)	219 (186-266)		211 (181-254)		230 (195-285)		<.001		
		ALP^j^ (U/L)	239 (195-295)		231 (190-284)		250 (203-312)		<.001		
	**Kidney function**										
		BUN^k^ (mg/dL)	16.0 (13.0-20.9)		15.3 (12.6-19.0)		17.9 (13.8-23.8)		<.001		
		Creatinine (mg/dL)	0.8 (0.6-1.0)		0.8 (0.7-1.0)		0.8 (0.6-1.1)		<.001		
		eGFR^l^ (mL/min/1.73 m^2^)	66.5 (51.2-81.5)		70.2 (55.9-83.8)		60.8 (44.8-76.5)		<.001		
	**Glycemic control**										
		Glucose (mg/100 mL)	121 (103-156)		119 (103-154)		124 (105-158)		.001		
		Hemoglobin A_1c_ (%)	5.9 (5.6-6.6)		5.9 (5.6-6.6)		5.9 (5.5-6.5)		<.001		
	**Inflammation**										
		hsCRP^m^, mg/dL	1.5 (0.5-6.1)		1.0 (0.4-2.9)		3.9 (1.0-16.3)		<.001		
	**Coagulation**										
		PT-INR^n^	1.0 (1.0-1.1)		1.0 (1.0-1.1)		1.1 (1.0-1.1)		<.001		
		APTT^o^ (s)	29.7 (27.2-32.7)		29.5 (27.1-32.4)		30.1 (27.3-33.3)		<.001		
		Fibrinogen (mg/dL)	304 (260-359)		297 (256-349)		315 (267-375)		<.001		
		d-dimer (μg/mL)	0.9 (0.4-2.0)		0.6 (0.2-1.2)		1.7 (0.9-4.0)		<.001		

^a^mRS: modified Rankin Scale.

^b^NIHSS: National Institutes of Health Stroke Scale.

^c^SBP: systolic blood pressure.

^d^DBP: diastolic blood pressure.

^e^WBC: white blood cell count.

^f^RBC: red blood cell count.

^g^AST: aspartate aminotransferase.

^h^ALT: alanine aminotransferase.

^i^LDH: lactate dehydrogenase.

^j^ALP: alkaline phosphatase.

^k^BUN: blood urea nitrogen.

^l^eGFR: estimated glomerular filtration rate.

^m^hsCRP: high-sensitivity C-reactive protein.

^n^PT-INR: international normalized ratio of prothrombin time.

^o^APTT: activated partial thromboplastin time.

### Assessment of Model Performance

AUROCs varied depending on study populations, whereas differences between the machine learning algorithms were minimal in the same study population and for the same outcome. The AUROCs of data-driven models based on machine learning were generally higher than those of item-based models for predicting both 3-month poor functional outcome and all-cause death (Table 2). Similarly, AUPRCs were generally higher in data-driven models than in item-based models for predicting both poor functional outcome and all-cause death (Table 3). Regarding the Brier score, the data-driven models performed better than the item-based models (Table 4).

**Table 2:** Area under the receiver operating characteristic curve for predicting unfavorable clinical outcomes at 3 months using item-based and data-driven models^a^.

			Item-based model, median (95% CI)		Data-driven models, median (95% CI)						
					RR^b^		LASSO^c^		RF^d^		XGBoost^e^
**Poor functional outcome**											
	Population 1 (n=3832)	0.83 (0.80-0.85)		0.86 (0.83-0.89)		0.86 (0.84-0.89)		0.86 (0.84-0.88)		0.86 (0.83-0.89)	
	Population 2 (n=6154)	0.88 (0.86-0.90)		0.91 (0.90-0.93)		0.91 (0.90-0.93)		0.91 (0.89-0.92)		0.91 (0.89-0.93)	
	Population 3 (n=6855)	0.87 (0.85-0.89)		0.90 (0.89-0.92)		0.90 (0.89-0.92)		0.90 (0.88-0.91)		0.90 (0.89-0.92)	
**Death**											
	Population 1 (n=3832)	0.77 (0.69-0.87)		0.87 (0.79-0.93)		0.87 (0.78-0.92)		0.89 (0.81-0.93)		0.88 (0.82-0.93)	
	Population 2 (n=6154)	0.84 (0.80-0.89)		0.89 (0.85-0.92)		0.88 (0.84-0.92)		0.90 (0.86-0.93)		0.90 (0.86-0.93)	
	Population 3 (n=6855)	0.82 (0.77-0.87)		0.88 (0.84-0.91)		0.87 (0.83-0.90)		0.89 (0.86-0.92)		0.89 (0.85-0.91)	

^a^The study populations were selected according to the inclusion and exclusion criteria for the Acute Stroke Registry and Analysis of Lausanne (ASTRAL) score (population 1), the preadmission comorbidities, level of consciousness, age, and neurologic deficit (PLAN) score (population 2), and the Ischemic Stroke Predictive Risk Score (iScore; population 3).

^b^RR: ridge regression.

^c^LASSO: least absolute shrinkage and selection operator regression.

^d^RF: random forest.

^e^XGBoost: Extreme Gradient Boosting.

**Table 3:** Area under the precision-recall curve for predicting unfavorable clinical outcomes at 3 months using item-based and data-driven models^a^.

		Item-based model, median (95% CI)	Data-driven models, median (95% CI)			
			RR^b^	LASSO^c^	RF^d^	XGBoost^e^
**Poor functional outcome**						
	Population 1 (n=3832)	0.71 (0.66-0.75)	0.75 (0.71-0.79)	0.75 (0.71-0.80)	0.74 (0.69-0.79)	0.75 (0.71-0.79)
	Population 2 (n=6154)	0.83 (0.80-0.86)	0.87 (0.85-0.89)	0.87 (0.85-0.90)	0.87 (0.84-0.89)	0.87 (0.85-0.89)
	Population 3 (n=6855)	0.83 (0.80-0.85)	0.87 (0.85-0.89)	0.87 (0.85-0.89)	0.86 (0.84-0.88)	0.87 (0.85-0.89)
**Death**						
	Population 1 (n=3832)	0.11 (0.06-0.24)	0.17 (0.08-0.32)	0.17 (0.07-0.31)	0.26 (0.13-0.44)	0.24 (0.12-0.39)
	Population 2 (n=6154)	0.17 (0.11-0.25)	0.27 (0.18-0.37)	0.27 (0.18-0.38)	0.29 (0.18-0.42)	0.27 (0.16-0.35)
	Population 3 (n=6855)	0.18 (0.11-0.25)	0.27 (0.16-0.36)	0.27 (0.17-0.38)	0.29 (0.19-0.42)	0.28 (0.19-0.39)

^a^The study populations were selected according to the inclusion and exclusion criteria for the Acute Stroke Registry and Analysis of Lausanne (ASTRAL) score (population 1), the preadmission comorbidities, level of consciousness, age, and Neurologic deficit (PLAN) score (population 2), and the Ischemic Stroke Predictive Risk Score (iScore; population 3).

^b^RR: ridge regression.

^c^LASSO: least absolute shrinkage and selection operator regression.

^d^RF: random forest.

^e^XGBoost: Extreme Gradient Boosting.

**Table 4:** Brier score for predicting unfavorable clinical outcomes at 3 months using item-based and data-driven models^a^.

		Item-based model, median (95% CI)	Data-driven models, median (95% CI)			
			RR^b^	LASSO^c^	RF^d^	XGBoost^e^
**Poor functional outcome**						
	Population 1 (n=3832)	0.15 (0.14-0.17)	0.14 (0.12-0.15)	0.14 (0.12-0.15)	0.14 (0.13-0.15)	0.14 (0.12-0.15)
	Population 2 (n=6154)	0.13 (0.12-0.14)	0.11 (0.10-0.12)	0.11 (0.10-0.12)	0.12 (0.11-0.13)	0.11 (0.10-0.12)
	Population 3 (n=6855)	0.13 (0.12-0.15)	0.12 (0.11-0.13)	0.12 (0.11-0.13)	0.12 (0.12-0.13)	0.12 (0.11-0.13)
**Death**						
	Population 1 (n=3832)	0.03 (0.02-0.03)	0.03 (0.02-0.03)	0.03 (0.02-0.03)	0.03 (0.02-0.03)	0.03 (0.02-0.03)
	Population 2 (n=6154)	0.03 (0.02-0.04)	0.03 (0.02-0.04)	0.03 (0.02-0.04)	0.03 (0.02-0.04)	0.03 (0.02-0.04)
	Population 3 (n=6855)	0.03 (0.02-0.04)	0.03 (0.02-0.04)	0.03 (0.02-0.04)	0.03 (0.02-0.04)	0.03 (0.02-0.04)

^a^The study populations were selected according to the inclusion and exclusion criteria for the Acute Stroke Registry and Analysis of Lausanne (ASTRAL) score (population 1), the preadmission comorbidities, level of consciousness, age, and neurologic deficit (PLAN) score (population 2), and the Ischemic Stroke Predictive Risk Score (iScore; population 3).

^b^RR: ridge regression.

^c^LASSO: least absolute shrinkage and selection operator regression.

^d^RF: random forest.

^e^XGBoost: Extreme Gradient Boosting.

The predictive performance of data-driven models compared with the corresponding item-based model was examined by the frequency of the performance metrics (AUROC, AUPRC, and Brier score) of data-driven models, which were better than those of the corresponding item-based model in the identical training and test data sets after 100 repeated random splits (Table 5). Regarding poor functional outcome, the frequency exceeded 95% for all metrics in all the data-driven models (RR, LASSO, RF, and XGBoost), indicating that the probability of the worse performance of data-driven models compared with the item-based model was <5%. Regarding death, the frequency was >95% for AUROC in all the data-driven models but did not always attain 95% for AUPRC or Brier score.

**Figure 3:**
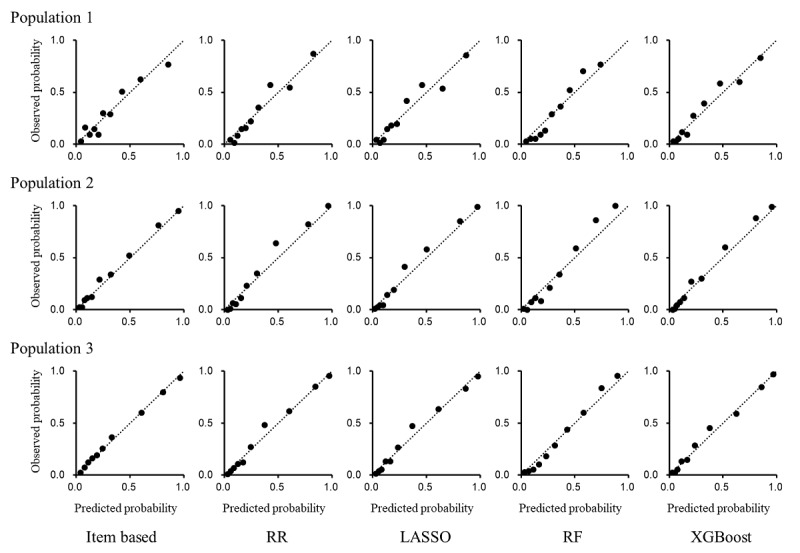
Calibration of item-based and data-driven models for predicting poor functional outcome. Calibration for predicting poor functional outcome was compared between the item-based regression model and data-driven models (ridge regression [RR], least absolute shrinkage and selection operator regression [LASSO], random forest [RF], and Extreme Gradient Boosting [XGBoost]) in population 1 for the Acute Stroke Registry and Analysis of Lausanne (ASTRAL) score, population 2 for the preadmission comorbidities, level of consciousness, age, and neurologic deficit (PLAN) score, and population 3 for the Ischemic Stroke Predictive Risk Score (iScore). The patients were categorized into 10 groups stratified by the predicted probability of poor functional outcome in the test data. Observed probabilities (x-axis) were plotted against predicted probabilities (y-axis) in the 10 groups based on risk stratification. The results for the first 100 random splits are presented.

**Figure 4:**
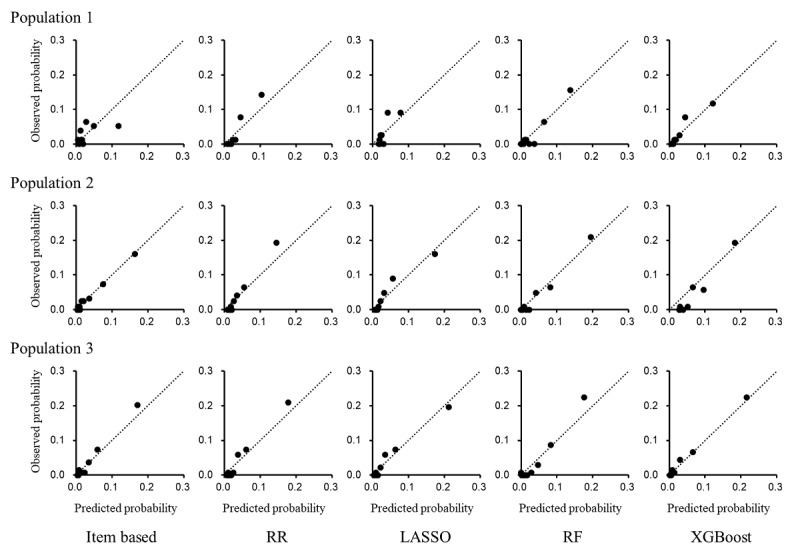
Calibration of item-based and data-driven models for predicting death. Calibration for predicting death was compared between the item-based regression model and data-driven models (ridge regression [RR], least absolute shrinkage and selection operator regression [LASSO], random forest [RF], and Extreme Gradient Boosting [XGBoost]) in population 1 for the Acute Stroke Registry and Analysis of Lausanne (ASTRAL) score, population 2 for the preadmission comorbidities, level of consciousness, age, and neurologic deficit (PLAN) score, and population 3 for the Ischemic Stroke Predictive Risk Score (iScore). The patients were categorized into 10 groups stratified by the predicted probability of death in the test data. Observed probabilities (x-axis) were plotted against predicted probabilities (y-axis) in the 10 groups based on risk stratification. The results for the first 100 random splits are presented.

Calibration for predicting poor functional outcome was compared between the item-based and data-driven models (RR, LASSO, RF, and XGBoost) in population 1 for the ASTRAL score, in population 2 for the PLAN score, and in population 3 for the iScore. The prediction of poor functional outcome (Figure 3) and all-cause death (Figure 4) demonstrated concordance between the predicted and observed probabilities in the item-based models as well as in the data-driven models.

**Table 5:** Predictive performance of data-driven models versus item-based models^a^.

			Poor functional outcome									Death						
			RR^b^		LASSO^c^		RF^d^		XGBoost^e^		RR			LASSO		RF		XGBoost
**AUROC^f^**																		
	Population 1 (n=3832)	100		100		100		100		97			95		97		96	
	Population 2 (n=6154)	100		100		100		100		100			100		98		99	
	Population 3 (n=6855)	100		100		100		100		100			99		100		99	
**AUPRC^g^**																		
	Population 1 (n=3832)	100		100		99		98		81			78		93		93	
	Population 2 (n=6154)	100		100		100		100		99			99		99		100	
	Population 3 (n=6855)	100		100		100		100		98			98		100		98	
**Bier score**																		
	Population 1 (n=3832)	100		100		99		100		83			70		96		89	
	Population 2 (n=6154)	100		100		100		100		98			92		97		93	
	Population 3 (n=6855)	100		100		100		100		100			99		100		96	

^a^Data indicate the frequency that AUROC, AUPRC, and Brier score of data-driven models (RR, LASSO, RF, or XGBoost) exceeded those of item-based models in identical training and test sets after 100 repeated random splits.

^b^RR: ridge regression.

^c^LASSO: least absolute shrinkage and selection operator regression.

^d^RF: random forest.

^e^XGBoost: Extreme Gradient Boosting.

^f^AUROC: area under the receiver operating characteristic curve.

^g^AUPRC: area under the precision-recall curve.

### Evaluation of Variables

**Figure 5:**
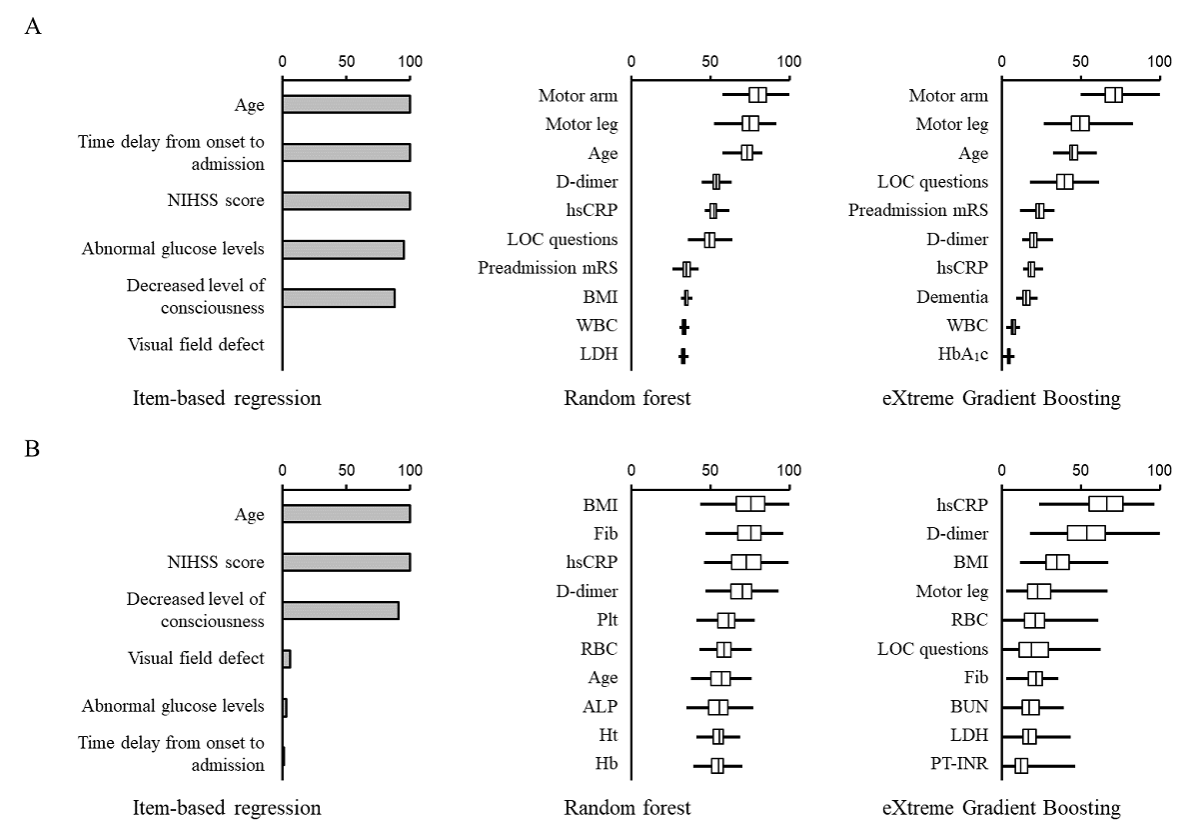
Comparison of variable importance between items of the Acute Stroke Registry and Analysis of Lausanne (ASTRAL) score and explanatory variables in machine learning model in population 1. The contribution of each variable to the models in predicting poor functional outcome (A) and death (B) is shown. The patients were selected based on the ASTRAL criteria (population 1). In item-based regression models, the percentage indicates the rate of times when its association with clinical outcomes was statistically significant (*P*<.05). In machine learning models, the top 10 variables are shown according to the magnitude of variable importance. Boxes, vertical lines in the boxes, and horizontal bars indicate IQR, median, and minimal or maximal range, respectively. NIHSS: National Institutes of Health Stroke Scale, hsCRP: high-sensitivity C-reactive protein, LOC: loss of consciousness, mRS: modified Rankin Scale, BMI: body mass index, WBC: white blood cell count, LDH: lactate dehydrogenase, HbA1c: hemoglobin A1c, Fib: fibrinogen, Plt: platelet count, RBC: red blood cell count, ALP: alkaline phosphatase, Ht: hematocrit, Hb: hemoglobin, BUN: blood urea nitrogen, LDH: lactate dehydrogenase, PT-INR: international normalized ratio of prothrombin time.

**Figure 6:**
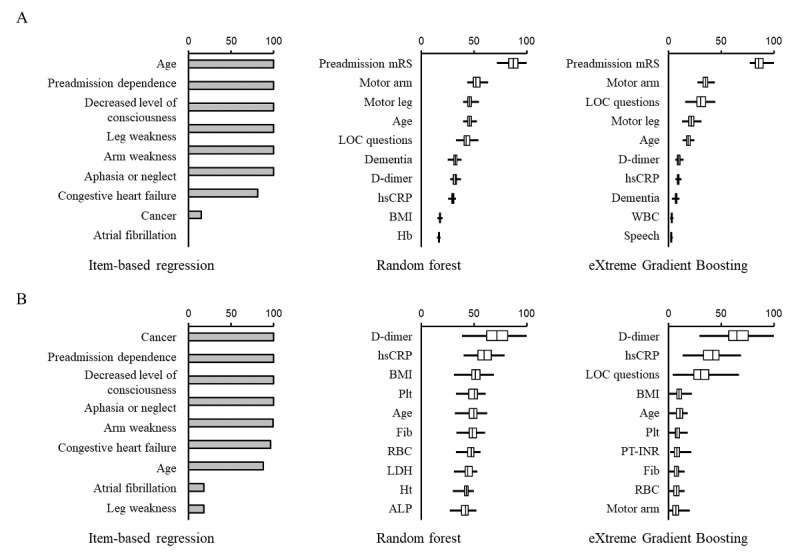
Comparison of variable importance between items of the preadmission comorbidities, level of consciousness, age, and neurologic deficit (PLAN) score and explanatory variables in machine learning model in population 2. The contribution of each variable to the models in predicting poor functional outcome (A) and death (B) is shown. The patients were selected based on the PLAN score criteria (population 2). In item-based regression models, the percentage indicates the rate of times when its association with clinical outcomes was statistically significant (*P*<.05). In machine learning models, the top 10 variables are shown according to the magnitude of variable importance. Boxes, vertical lines in the boxes, and horizontal bars indicate IQR, median, and minimal or maximal range, respectively. mRS: modified Rankin Scale, LOC: loss of consciousness, hsCRP: high-sensitivity C-reactive protein, BMI: body mass index, Hb: hemoglobin, WBC: white blood cell count, Plt: platelet count, Fib: fibrinogen, RBC: red blood cell count, LDH: lactate dehydrogenase, Ht: hematocrit, ALP: alkaline phosphatase, PT-INR: international normalized ratio of prothrombin time.

**Figure 7:**
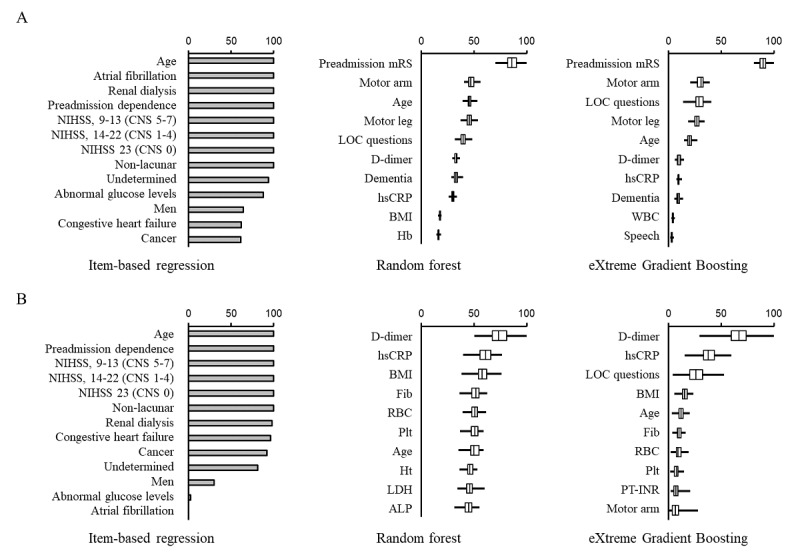
Comparison of variable importance between items of Ischemic Stroke Predictive Risk Score (iScore) and explanatory variables in machine learning model in population 3. The contribution of each variable to the models in predicting poor functional outcome (A) and death (B) is shown. The patients were selected according to the iScore criteria (population 3). In item-based regression models, the percentage indicates the rate of times when its association with clinical outcomes was statistically significant (*P*<.05). In machine learning models, the top 10 variables are shown according to the magnitude of variable importance. Boxes, vertical lines in the boxes, and horizontal bars indicate IQR, median, and minimal or maximal range, respectively. NIHSS: National Institutes of Health Stroke Scale, CNS: Canadian Neurological Scale, mRS: modified Rankin Scale, LOC: loss of consciousness, hsCRP: high-sensitivity C-reactive protein, BMI: body mass index, Hb: hemoglobin, WBC: white blood cell count, Fib: fibrinogen, RBC: red blood cell count, Plt: platelet count, Ht: hematocrit, LDH: lactate dehydrogenase, ALP: alkaline phosphatase, PT-INR: international normalized ratio of prothrombin time.

Next, we evaluated how each variable contributed to the predictive performance of the item-based and data-driven models (RF and XGBoost) in population 1 (Figure 5), population 2 (Figure 6), and population 3 (Figure 7). The selected variables differed substantially between the study populations in the item-based models. Age, preadmission dependence, and neurological severity of stroke were important variables in predicting both poor functional outcome and death (Figures 5-7; left panels). Age and neurological deficit signs (arm or leg weakness and loss of consciousness) were the most frequently used variables for predicting poor functional outcome (Figures 5A, 6A, and 7A; middle and right panels) in RF and XGBoost. In contrast, variables not used in the item-based models, such as d-dimer, high-sensitivity C-reactive protein, fibrinogen, and BMI, were the most frequently used variables by RF and XGBoost (Figures 5B, 6B, and 7B; middle and right panels) in predicting death.

**Figure 8:**
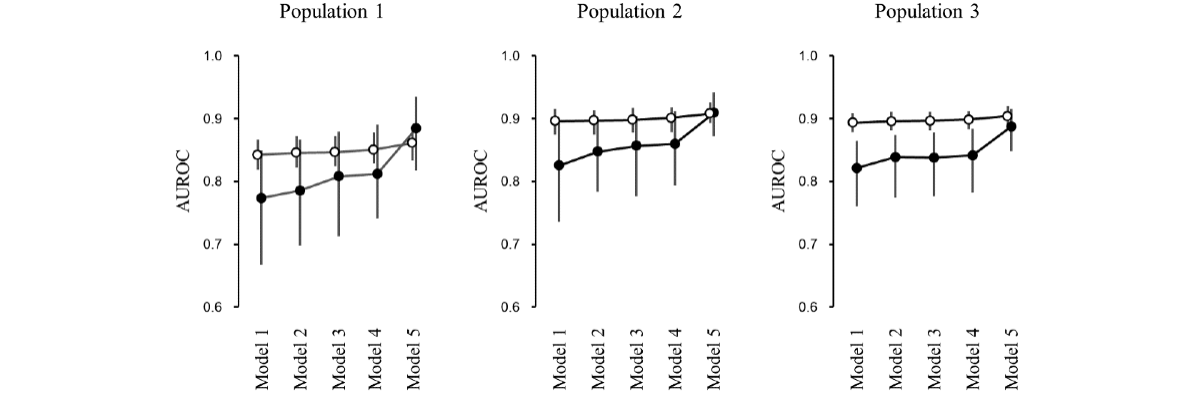
Improvement of discrimination in a data-driven model by adding different types of data. The area under the receiver operating characteristic curves (AUROCs) for predicting poor functional outcome (open circles) and death (closed circles) were compared among the 5 models, which used different types of variables. A data-driven model was developed for each population using Extreme Gradient Boosting. Vertical bars indicate the 95th percentile after 100 random splits. The variables used for the models were as follows: model 1: age, sex, National Institutes of Health Stroke Scale score, and preadmission modified Rankin Scale score; model 2: model 1 plus clinical data before admission (eg, risk factors, comorbid conditions, previous history, family history, and prestroke medication); model 3: model 2 plus clinical data on admission (eg, onset-to-admission time, ambulance use, BMI, and physiological data); model 4: model 3 plus brain imaging data (eg, site of lesion, side of lesion, and stroke subtype); and model 5: model 4 plus laboratory data.

We also investigated how the addition of variables increased the predictive performance of XGBoost. As a result, the AUROC for poor functional outcome did not substantially increase even when explanatory variables other than key predictors were added to model 1 (Figure 8; open circles). Conversely, the AUROC for all-cause death linearly increased with the addition of other variables to the models, particularly items from laboratory data (Figure 8; closed circles).

## Discussion

### Principal Findings

This study, which analyzed comprehensive clinical data from a multicenter, hospital-based stroke registry, yielded the following major findings. The performance of item-based regression models using the predictors of 3 conventional stroke prognostic scores was fair in predicting clinical outcomes at 3 months after ischemic stroke in our cohort, despite differences in clinical and social backgrounds from the original cohorts of scores. Data-driven models based on machine learning algorithms exhibited better performance when compared with item-based models in identical study populations. The importance of variables in RF and XGBoost appeared to differ from that in item-based models when predicting death within 3 months. The addition of nonconventional factors, such as laboratory data, to the XGBoost model improved its predictive ability for 3-month mortality.

### Predictive Performance of Models

Thus far, only a limited number of studies have evaluated the predictive performance of machine learning–based models compared with those of stroke prognostic scores [19,20,23]. All these studies were performed in single-center registries or under specific conditions, such as large vessel occlusion in ischemic stroke. Furthermore, previous studies mainly focused on AUROC for assessing predictive performance, although other metrics, such as measures of calibration, are necessary to fully evaluate the performance of models [38]. This study was conducted using a multicenter registry database and several performance metrics. Our study demonstrated that data-driven models developed using machine learning algorithms can perform reasonably well in predicting the 3-month clinical outcomes of patients with acute ischemic stroke. Generally, data-driven models performed better than conventional prognostic scores when both were compared in identical study populations.

This study also demonstrates that the model performance largely depends on the study populations. The study populations varied in terms of both size and patient characteristics, such as prestroke dependency, time from onset to admission, and use of thrombolytic therapy. The variability in AUROC, AUPRC, and Brier scores between the study populations was as large as that between the models. Moreover, the model performance varied depending on the outcomes to be predicted: AUPRCs were substantially decreased for the prediction of death, which is a less frequent event than the poor functional outcome. These findings underscore the reiterated importance of sample size, the number of outcome events, and data quality of the study cohorts where models are to be developed and validated [25,39,40].

### Variables in Models

In this study, age, preadmission dependence, and variables related to neurological deficits were identified as important predictors for the prediction of poor functional outcome in both item-based regression models and data-driven models using RF and XGBoost. These are well-known risk factors for poor functional outcome and are also used for predicting death in stroke prognostic scores [4,5,7]. However, BMI and items related to laboratory data, such as D-dimer, high-sensitivity C-reactive protein, and fibrinogen, were found to be the most important variables for predicting death in RF and XGBoost. Indeed, the association between poststroke clinical outcomes and markers of inflammation and hypercoagulation has become a recent research topic [41,42]. Machine learning algorithms can be a promising tool to identify novel factors to be considered in making prognoses for stroke because they can maximize the use of data without arbitrary assumptions and procedures.

### Clinical Implications

The ability of machine learning to derive a model that best fits the data on a given cohort is appealing for making prognoses. Prognostic scores with prespecified items may not fit all cohorts because heterogeneity must exist between study cohorts in race or ethnic groups, general health conditions, socioeconomic status, and health care systems. In addition, stroke prognostic scores are at risk of getting outdated over time, as advances in stroke care continuously improve clinical outcomes in patients with stroke [43,44]. However, our analysis suggests that the 3 conventional prognostic scores can perform sufficiently well in our cohort, despite the fact that the original studies that developed the scores had patients with different medical backgrounds and during different study periods. This finding demonstrates the robustness of outcome prediction using regression models in terms of generalizability. Furthermore, considering nonlinear and interaction effects might not be crucial for outcome prediction after ischemic stroke, as the simple regression models worked well in our study.

Point-based stroke prognostic scores are convenient and helpful for making prompt decisions at the bedside. Generally, prognostic scores comprise only a handful of variables on which information can be obtained easily. This advantage in the practicability of the prognostic scores is important in acute stroke care settings. Machine learning algorithms require more data than conventional prognostic scores to reach acceptable performance levels [39], and the data required by machine learning algorithms to realize better performance, such as laboratory data, may not always be available, although they can improve the predictive performance of models. Therefore, further studies are needed to fully assess the incremental value of machine learning–based models in daily clinical practice.

### Strengths and Limitations

This study has several strengths. We assessed and compared the predictive accuracy of prognostic scores against data-driven models, using information from a multicenter, prospective registry of individuals diagnosed with acute stroke. We were able to use several variables, including laboratory data–related items, owing to the detailed clinical data available in the registry. Moreover, comparisons of models were made using various performance metrics. However, this study has also several limitations. First, the selection of patients may have led to bias, although the inclusion and exclusion criteria were identical to those reported in the original studies of the prognostic scores. Second, there were missing data for the baseline variables and clinical outcomes, which may have also led to selection bias. Third, the possibility of overfitting cannot be completely ruled out, despite the predictive models constituted by the training set being fitted to the test set. Finally, this study included only patients with acute ischemic stroke who were hospitalized in tertiary care centers in a restricted region of Japan. Generalizability should be assessed in other settings and for other diseases.

### Conclusions

This study suggests that data-driven models based on machine learning algorithms can improve predictive performance by using diverse types of variables, such as laboratory data–related items. The clinical outcomes of individual patients can be automatically estimated using machine learning algorithms if a large amount of data can be directly drawn from electronic health records. This possibility of making automated and personalized prognoses is an appealing property of data-driven prediction. However, the arrangement of an appropriate electronic infrastructure is indispensable for enabling data collection, and the development of such infrastructure requires time and cost. It is worth noting that conventional prognostic scores can achieve sufficient performance in making stroke prognoses with only a limited number of variables. In the near future, it seems feasible to explore the improvement of preexisting prognostic scores by incorporating novel predictors identified by machine learning algorithms, given the significant investment necessary to fully use machine learning.

## Appendix

Multimedia Appendix 1 Stroke prognostic scores.

Multimedia Appendix 2 Study populations and events based on the criteria of stroke prognostic scores.

Multimedia Appendix 3 Rates of missing values.

Multimedia Appendix 4 R programs for the development of machine learning–based models.

Multimedia Appendix 5 Baseline data according to death within 3 months.

## Abbreviations

ASTRAL: Acute Stroke Registry and Analysis of Lausanne
AUPRC: area under the precision-recall curve
AUROC: area under the receiver operating characteristic curve
FSR: Fukuoka Stroke Registry
iScore: Ischemic Stroke Predictive Risk Score
LASSO: least absolute shrinkage and selection operator
PLAN: preadmission comorbidities, level of consciousness, age, and neurologic deficit
RF: random forest
RR: ridge regression
TRIPOD: Transparent Reporting of a Multivariable Prediction Model for Individual Prognosis or Diagnosis
XGBoost: Extreme gradient boosting
