# Supplementary material for: Predictive Performance of Machine Learning–Based Models for Poststroke Clinical Outcomes in Comparison With Conventional Prognostic Scores: Multicenter, Hospital-Based Observational Study
Source: JMIR AI. 2024 Jan 11;3:e46840. doi: 10.2196/46840 (PMC11041492; doi:10.2196/46840)
Supplement: Multimedia Appendix 1 [file ai_v3i1e46840_app1.docx]

Appendix 1

**Stroke prognostic scores**

| Predictors | ASTRAL | PLAN | iScore |
| --- | --- | --- | --- |
|  |  |  |  |
| **Demographics** |  |  |  |
| Age | 1 pt /5 y | 1 pt /10 y | 1pt /y |
| Men |  |  | 10 pts |
| **Risk factors** |  |  |  |
| Atrial fibrillation |  | 1 pt | 10 pts |
| **Comorbid conditions** |  |  |  |
| Congestive heart failure |  | 1 pt | 10 pts |
| Renal dialysis |  |  | 35 pts |
| Cancer |  | 1.5 pts | 10 pts |
| **Preadmission functional status** |  |  |  |
| Preadmission dependence |  | 1.5 pts | 15 pts |
| **Onset-to-admission time** |  |  |  |
| Time delay from onset to admission | 2 pts |  |  |
| **Neurological severity** |  |  |  |
| Stroke scale score | 1 pt /score |  |  |
| NIHSS, 9–13 (CNS 5–7) |  |  | 40 pts |
| NIHSS, 14–22 (CNS 1–4) |  |  | 65 pts |
| NIHSS 23 (CNS 0) |  |  | 105 pts |
| **Neurological deficit** |  |  |  |
| Decreased level of consciousness | 3 pts | 5 pts |  |
| Leg weakness |  | 2 pts |  |
| Arm weakness |  | 2 pts |  |
| Aphasia or neglect |  | 1 pt |  |
| Visual field defect | 2 pts |  |  |
| **Stroke subtype** |  |  |  |
| Non-lacunar |  |  | 30 pts |
| Undetermined |  |  | 35 pts |
| **Laboratory data** |  |  |  |
| Abnormal glucose levels | 1 pt |  | 15 pts |

Late admission was defined as admission more than 24 h after stroke onset. The time delay from onset to admission was defined as an onset-to-admission time >3 h. The CNS scores were converted to NIHSS scores according to a previously reported conversion formula because iScore assessed neurological severity based on CNS. Abnormal glucose levels were defined as >7.3 mmol/L (132 mg/dL) or <3.7 mmol/L (66 mg/dL) for the ASTRAL score and ≥7.5 mmol/L (135 mg/dL) for the iScore. Preadmission dependence was defined as a modified Rankin Scale score >2 for the ASTRAL score and a modified Rankin Scale score >1 for the PLAN and iScore scores.
