# Supplementary material for: Predictive Performance of Machine Learning–Based Models for Poststroke Clinical Outcomes in Comparison With Conventional Prognostic Scores: Multicenter, Hospital-Based Observational Study
Source: JMIR AI. 2024 Jan 11;3:e46840. doi: 10.2196/46840 (PMC11041492; doi:10.2196/46840)
Supplement: Multimedia Appendix 2 [file ai_v3i1e46840_app2.docx]

# Appendix 2

**Study populations and events based on criteria of stroke prognostic scores**

|  | Population 1  ASTRAL | Population 2  PLAN | Population 3  iScore |
| --- | --- | --- | --- |
|  |  |  |  |
| **Study population** |  |  |  |
| Age <18 years |  | Excl. | Excl. |
| Transient ischemic attack | Excl. | Excl. | Excl. |
| Preadmission dependence | Excl. |  |  |
| Late admission | Excl. |  |  |
| Thrombolytic therapy |  | Excl. |  |
| **Sample size** |  |  |  |
| Number of patients, n | 3832 | 6154 | 6855 |
| **Event** |  |  |  |
| Poor functional outcome, n (%) | 1204 (31.4) | 2209 (35.9) | 2540 (37.1) |
| Death, n (%) | 113 (3.0) | 219 (3.6) | 255 (3.7) |

Population 1, population 2, and population 3 were selected according to the inclusion and exclusion criteria of the ASTRAL score, PLAN score, and iScore.

ka, Japan).
