# Supplementary material for: Predictive Performance of Machine Learning–Based Models for Poststroke Clinical Outcomes in Comparison With Conventional Prognostic Scores: Multicenter, Hospital-Based Observational Study
Source: JMIR AI. 2024 Jan 11;3:e46840. doi: 10.2196/46840 (PMC11041492; doi:10.2196/46840)
Supplement: Multimedia Appendix 3 [file ai_v3i1e46840_app3.docx]

# Appendix 3

**Rates of missing values**

| **Demographics** |  | **Neurological severity** |  |
| --- | --- | --- | --- |
| Age | 0.0 | NIHSS score | 0.0 |
| Men | 0.0 | **Neurological deficit** |  |
| **Risk factors** |  | Score for each item of NIHSS | 2.3 |
| Hypertension | 0.0 | **Physiological data** |  |
| Diabetes mellitus | 0.1 | Systolic blood pressure | 0.0 |
| Atrial fibrillation | 0.1 | Diastolic blood pressure | 0.0 |
| Dyslipidemia | 0.0 | Heart rate | 0.0 |
| Smoking | 0.2 | Body mass index | 0.1 |
| Drinking | 0.1 | Waist circumference | 1.7 |
| **Comorbid conditions** |  | **Brain imaging** |  |
| Congestive heart failure | 0.0 | Diffusion-weighted image finding | 0.0 |
| Kidney disease on dialysis | 0.0 | Side of lesion | 0.7 |
| End-stage renal failure | 0.0 | Site of lesion | 0.0 |
| Cancer | 0.0 | **Laboratory data** |  |
| Allergy | 2.0 | White blood cell count | 0.0 |
| Arteriosclerosis obliterans | 10.0 | Red blood cell count | 0.0 |
| Valvular disease | 0.3 | Hematocrit | 0.0 |
| Ischemic heart disease | 0.3 | Hemoglobin | 0.0 |
| Cardiomyopathy | 0.1 | Platelet count | 0.0 |
| Dementia | 1.9 | Aspartate aminotransferase | 0.0 |
| **Previous history** |  | Alanine aminotransferase | 0.0 |
| Stroke | 0.3 | Lactate dehydrogenase | 0.6 |
| Other cardiovascular diseases | 1.4 | Alkaline phosphatase | 1.7 |
| **Family history** |  | Total bilirubin | 0.7 |
| Stroke | 3.4 | Low-density lipoprotein cholesterol | 4.4 |
| **Preadmission functional status** |  | High-density lipoprotein cholesterol | 1.8 |
| Preadmission mRS | 0.0 | Triglycerides | 1.5 |
| **Prestroke medication** |  | Total protein | 0.7 |
| Anticoagulants | 0.0 | Creatine phosphokinase | 5.7 |
| Antiplatelets | 0.0 | Blood urea nitrogen | 0.0 |
| Antidyslipidemic drugs | 0.0 | Creatinine | 0.0 |
| Antihypertensives | 0.0 | Estimated glomerular filtration rate | 0.0 |
| Antidiabetic drugs | 0.0 | Glucose | 0.4 |
| **Date of onset** |  | Hemoglobin A1c | 0.5 |
| Day of week | 0.0 | Sodium | 0.3 |
| Month | 0.0 | Potassium | 0.3 |
| **Time from onset to admission** |  | High-sensitivity C-reactive protein | 2.1 |
| Onset-to-admission time | 0.0 | PT-INR | 0.7 |
| **Transport to hospital** |  | APTT | 0.8 |
| Ambulance use | 0.0 | Fibrinogen | 2.2 |
| **Stroke etiology** |  | D-dimer | 11.3 |
| Stroke subtype | 0.0 |  |  |

Rates of missing values are shown for all variables used in the data-driven models.
