# Supplementary material for: Predictive Performance of Machine Learning–Based Models for Poststroke Clinical Outcomes in Comparison With Conventional Prognostic Scores: Multicenter, Hospital-Based Observational Study
Source: JMIR AI. 2024 Jan 11;3:e46840. doi: 10.2196/46840 (PMC11041492; doi:10.2196/46840)
Supplement: Multimedia Appendix 4 [file ai_v3i1e46840_app4.docx]

# Appendix 4

## R programs for the development of machine learning-based models

The data were analyzed using RStudio (http://www.rstudio. com/, version 1.4.1106) with the R statistical package (http://www.r-project.org/, version 4.0.4/4.1.0).

In ridge and Least Absolute Shrinkage and Selection Operator (LASSO) regression, we used the R program “glmnet (https://cran.r-project.org/web/packages/glmnet/glmnet.pdf)” and tuned parameter λ after increasing by 0.001 between 0 and 1 using 10-fold cross-validation with Grid search using “caret (https://cran.r-project.org/web/packages/caret/caret.pdf).” We used the R programs “randomForest (https://cran.r-project.org/web/packages/randomForest/randomForest.pdf)” and “xgboost (https://cran.r-project.org/web/packages/xgboost/xgboost.pdf)” for random forest and eXtreme Gradient Boosting, respectively, and “caret” to tune their parameters. In random forest, “mtry,” the number of variables that were randomly sampled as candidates at each split, was tuned between 1 and 11. The maximum number 11 was obtained by the square root of the number of all explanatory variables. In eXtreme Gradient Boosting, we tuned “nrounds,” the maximum number of boosting iterations between 1 and 100, and “max_depth,” the maximum depth of a tree between 1 and 10, by 10-fold cross-validation with Grid search. The other parameters were fixed as follows: “eta,” 0.1; “gamma,” 0; “colsample_bytree,” 1; “min_child_weight,” 1; and “subsample,” 1.

The importance of variables was evaluated using the mean decrease in node impurity in the random forest model and Gain in the eXtreme Gradient Boosting model.
