# Supplementary material for: Predictive Performance of Machine Learning–Based Models for Poststroke Clinical Outcomes in Comparison With Conventional Prognostic Scores: Multicenter, Hospital-Based Observational Study
Source: JMIR AI. 2024 Jan 11;3:e46840. doi: 10.2196/46840 (PMC11041492; doi:10.2196/46840)
Supplement: Multimedia Appendix 5 [file ai_v3i1e46840_app5.docx]

# Appendix 5

**Baseline data according to death within 3 months**

|  | Survival, n=10055 | Death, n=458 | ***P*** |
| --- | --- | --- | --- |
|  |  |  |  |
| **Demographics** |  |  |  |
| Age, year | 72.6 (12.4) | 81.6 (10.5) | <.001 |
| Men | 5988 (59.6) | 221 (48.3) | <.001 |
| **Risk factors** |  |  |  |
| Hypertension | 8133 (80.9) | 352 (77.2) | 0.06 |
| Diabetes mellitus | 3474 (34.6) | 133 (29.0) | 0.02 |
| Atrial fibrillation | 2507 (25.0) | 236 (51.6) | <.001 |
| Smoking | 2219 (23.7) | 42 (10.0) | <.001 |
| **Comorbid conditions** |  |  |  |
| Congestive heart failure | 840 (8.4) | 79 (17.3) | <.001 |
| Kidney disease on dialysis | 300 (3.0) | 32 (7.0) | <.001 |
| Cancer | 1431 (14.2) | 121 (26.4) | <.001 |
| **Previous history** |  |  |  |
| Previous myocardial infarction | 469 (5.2) | 36 (8.2) | 0.009 |
| **Preadmission functional status** |  |  |  |
| Preadmission mRS | 0 (0–1) | 2 (0–4) | <.001 |
| Preadmission dependence | 2118 (21.1) | 248 (54.2) | <.001 |
| **Onset-to-admission time** |  |  | <.001 |
| ≤1h | 872 (8.7) | 71 (15.5) |  |
| ≤3h | 1373 (13.7) | 96 (21.0) |  |
| ≤6h | 1079 (10.7) | 62 (13.5) |  |
| ≤24h | 3362 (33.4) | 153 (33.4) |  |
| 24h< | 3369 (33.5) | 76 (16.6) |  |
| **Stroke subtype** |  |  | <.001 |
| Small vessel occlusion | 2104 (20.9) | 15 (3.3) |  |
| Large artery atherosclerosis | 1759 (17.5) | 64 (14.0) |  |
| Cardioembolism | 2262 (22.5) | 234 (51.1) |  |
| Other determined etiology | 2076 (20.7) | 70 (15.3) |  |
| Undetermined | 1854 (18.4) | 75 (16.4) |  |
| **Neurological severity** |  |  |  |
| NIHSS score | 3 (2–7) | 15 (5–22) | <.001 |
| Severe stroke | 1656 (16.5) | 282 (61.6) | <.001 |
| **Neurological deficits** |  |  |  |
| Decreased level of consciousness | 2790 (28.0) | 339 (74.7) | <.001 |
| Leg weakness | 5032 (50.6) | 362 (80.6) | <.001 |
| Arm weakness | 5257 (52.8) | 377 (83.6) | <.001 |
| Aphasia or neglect | 2604 (26.1) | 308 (67.8) | <.001 |
| Visual field defect | 913 (9.2) | 86 (19.0) | <.001 |
| **Physiological data** |  |  |  |
| SBP, mmHg | 86.8 (18.0) | 82.5 (20.5) | <.001 |
| DBP, mmHg | 160.1 (29.2) | 152.1 (30.2) | <.001 |
| BMI, kg/m^2^ | 22.9 (3.8) | 20.3 (3.7) | <.001 |
| **Laboratory data** |  |  |  |
| **Complete blood cell count** |  |  |  |
| WBC, 10^3^/μL | 6.8 (5.6–8.4) | 7.3 (5.8–9.9) | <.001 |
| RBC, 10^4^/μL | 438 (397–477) | 386 (343–433) | <.001 |
| Ht, % | 40.3 (36.7–43.5) | 35.8 (31.8–39.8) | <.001 |
| Hb, g/dL | 13.6 (12.2–14.8) | 11.8 (10.4–13.4) | <.001 |
| Plt, 10^4^/μL | 20.3 (16.7–24.4) | 18.0 (13.7–22.9) | <.001 |
| **Liver function** |  |  |  |
| AST, U/L | 23 (19–29) | 25 (19–34) | <.001 |
| ALT, U/L | 17 (12–24) | 14 (10–23) | <.001 |
| LDH, U/L | 218 (185–264) | 254 (205–338) | <.001 |
| ALP, U/L | 237 (194–294) | 260 (208–327) | <.001 |
| **Kidney function** |  |  |  |
| BUN, mg/dL | 16.0 (13.0–20.4) | 20.3 (15.6–28.5) | <.001 |
| Cr, mg/dL | 0.8 (0.6–1.0) | 0.9 (0.7–1.2) | <.001 |
| eGFR, mL/min/1.73m^2^ | 67.1 (51.2–81.8) | 54.2 (36.1–71.5) | <.001 |
| **Glycemic control** |  |  |  |
| Glu, mg/dL | 121 (103–155) | 127 (107–164) | 0.02 |
| HbA1c, % | 5.9 (5.6–6.6) | 5.8 (5.5–6.3) | <.001 |
| **Inflammation** |  |  |  |
| hsCRP, mg/dL | 1.4 (0.5–5.4) | 11.5 (3.2–39.8) | <.001 |
| **Coagulation** |  |  |  |
| PT-INR | 1.0 (1.0–1.1) | 1.1 (1.0–1.2) | <.001 |
| APTT, sec | 29.6 (27.1–32.7) | 30.9 (27.7–34.1) | <.001 |
| Fib, mg/dL | 303 (260–358) | 318 (254–387) | 0.05 |
| D-dimer, μg/mL | 0.8 (0.4–1.9) | 4.0 (1.8–10.3) | <.001 |

Data are expressed as mean (standard deviation), median (interquartile range), or number (%). Preadmission dependence was defined as an mRS score >1 before admission. Severe stroke was defined as an NIHSS score >10.
